# Single-cell RNA sequencing identifies a novel proliferation cell type affecting clinical outcome of pancreatic ductal adenocarcinoma

**DOI:** 10.3389/fonc.2023.1236435

**Published:** 2023-08-02

**Authors:** Bicheng Ye, Qi Wang, Xiaofeng Zhu, Lingling Zeng, Huiyuan Luo, Yan Xiong, Qin Li, Qinmei Zhu, Songyun Zhao, Ting Chen, Jingen Xie

**Affiliations:** ^1^ Medical School, Yangzhou Polytechnic College, Yangzhou, China; ^2^ Department of Gastroenterology, Affiliated Hospital of Jiangsu University, Zhenjiang, China; ^3^ Department of Neurology, The Affiliated Huaian No.1 People’s Hospital of Nanjing Medical University, Huai’an, China; ^4^ Department of Gastroenterology, The Affiliated Huai’an Hospital of Xuzhou Medical University, The Second People’s Hospital of Huai’an, Huai’an, China; ^5^ Department of Neurosurgery, Wuxi People’s Hospital Affiliated to Nanjing Medical University, Wuxi, China; ^6^ Department of Oncology, The Affiliated Huai’an Hospital of Xuzhou Medical University, The Second People’s Hospital of Huai’an, Huai’an, China; ^7^ Department of General Medicine, Huai’an Cancer Hospital, Huai’an, China

**Keywords:** pancreatic ductal adenocarcinoma, single-cell sequencing, spatial transcriptome, proliferative cells, TP53, KRAS, immunotherapy

## Abstract

**Background:**

Pancreatic ductal adenocarcinoma (PDAC) is an extremely deadly neoplasm, with only a 5-year survival rate of around 9%. The tumor and its microenvironment are highly heterogeneous, and it is still unknown which cell types influence patient outcomes.

**Methods:**

We used single-cell RNA sequencing (scRNA-seq) and spatial transcriptome (ST) to identify differences in cell types. We then applied the scRNA-seq data to decompose the cell types in bulk RNA sequencing (bulk RNA-seq) data from the Cancer Genome Atlas (TCGA) cohort. We employed unbiased machine learning integration algorithms to develop a prognosis signature based on cell type makers. Lastly, we verified the differential expression of the key gene LY6D using immunohistochemistry and qRT-PCR.

**Results:**

In this study, we identified a novel cell type with high proliferative capacity, Prol, enriched with cell cycle and mitosis genes. We observed that the proportion of Prol cells was significantly increased in PDAC, and Prol cells were associated with reduced overall survival (OS) and progression-free survival (PFS). Additionally, the marker genes of Prol cell type, identified from scRNA-seq data, were upregulated and associated with poor prognosis in the bulk RNA-seq data. We further confirmed that mutant KRAS and TP53 were associated with an increased abundance of Prol cells and that these cells were associated with an immunosuppressive and cold tumor microenvironment in PDAC. ST determined the spatial location of Prol cells. Additionally, patients with a lower proportion of Prol cells in PDAC may benefit more from immunotherapy and gemcitabine treatment. Furthermore, we employed unbiased machine learning integration algorithms to develop a Prol signature that can precisely quantify the abundance of Prol cells and accurately predict prognosis. Finally, we confirmed that the LY6D protein and mRNA expression were markedly higher in pancreatic cancer than in normal pancreatic tissue.

**Conclusions:**

In summary, by integrating bulk RNA-seq and scRNA-seq, we identified a novel proliferative cell type, Prol, which influences the OS and PFS of PDAC patients.

## Introduction

Pancreatic ductal adenocarcinoma (PDAC) is a highly lethal cancer characterized by aggressive tumor growth, frequent delays in diagnosis, and poor prognosis, with a 5-year survival rate of only around 9% (1). Despite the use of surgical resection, chemotherapy, and antivascular therapy, the efficacy of these treatments remains limited (2, 3). One possible explanation for the different treatment responses and varying survival outcomes is the tumor heterogeneity of PDAC (4, 5). Current studies based on group transcriptome sequencing have encountered difficulties in explaining patient heterogeneity. Therefore, gaining a deeper understanding of the molecular and cellular characteristics of PDAC may provide valuable insights for identifying biomarkers and potential treatment options.

Single-cell RNA sequencing (scRNA-seq) is a potent and innovative technology that enables the acquisition of transcriptome data from single cells (6). By leveraging the high-throughput capabilities of this approach, researchers can accurately characterize the cell populations present within tumor tissue, which might otherwise be obscured by the dominant cell populations when utilizing traditional bulk sequencing techniques (7). As scRNA-seq methods continue to advance, their utilization for analyzing solid tissue, such as the PDAC, has become increasingly prevalent (7, 8). However, the identification of rare cell subpopulations still presents a significant challenge. To overcome this, it is imperative to integrate data from multiple scRNA-seq datasets, as this will enhance our capability to accurately uncover these rare cell populations.

Our hypothesis proposed that the integration of various scRNA-seq cohorts could uncover previously undetermined cell types associated with PDAC, and their potential effect on patient survival when their abundance increases within the tumor microenvironment (TME). In this study, we identified and characterized a highly proliferative PDAC cell type, called “Prol”, and showed that its abundance in tumors is associated with poor survival outcomes. KRAS and TP53 are major driver oncogenes in PDAC, with KRAS mutations in 90–95% and TP53 mutations in up to 75% of cases (9–11). KRAS is a membrane-bound protein that transmits growth factor signals, and its mutations lock it in an active state, leading to uncontrolled cell proliferation and survival (12). TP53 is a tumor suppressor gene that controls cell cycle, apoptosis, DNA repair, and other processes to prevent cancer. Its mutations impair its normal function and may confer gain-of-function properties that enhance tumor progression and metastasis (13). However, the relationship between these somatic mutations and cell type changes in PDAC is unclear. To fill this knowledge gap and reveal the molecular mechanisms of the identified cell types, we examined the PDAC risk cell type, Prol, for the accumulation of known somatic cancer mutations. In addition, machine learning offers advantages over traditional modeling by enabling the discovery of novel and informative features, using flexible and expressive functions, and combining different algorithms to improve prediction and classification (14). Thus, we successfully applied unbiased machine learning algorithms to create a novel Prol signature that can accurately quantify the abundance of Prol cells and predict clinical outcomes in PDAC patients. Our study may reveal new aspects of the pathophysiology of PDAC, which could lead to more personalized and beneficial treatments for patients with this cancer.

## Methods

### Analysis of the scRNA-seq data

We obtained two PDAC scRNA-seq datasets and extracted data of primary PDAC and non-tumor pancreatic tissue (15, 16), consisting of 34 samples from primary PDAC tumors and 11 samples from non-tumor tissue. We applied filters to the gene-cell matrix to eliminate cells with the number of features below 200 or greater than 8000 and mitochondrial genes exceeding 10%, and the count of captured transcripts exceeding 50000. A total of 73,415 cells were selected and imported into the Seurat (version 4.2.1) R package for subsequent analysis (17). To normalize the gene expression levels, we employed the LogNormalize method with a scale factor of 10,000. Next, we identified the 2000 most variable genes and scaled their expression levels before conducting principal component analysis (PCA) in variable gene space. The Harmony (version 0.1.1) R package was used to mitigate batch effects (18). All steps were carried out using functions from the Harmony and Seurat packages, including NormalizeData, FindVariableFeatures, ScaleData, RunPCA, FindNeighbors, FindClusters, and RunUMAP. We also performed the clustering of Prol cells following the aforementioned process. After obtaining 14 assigned clusters using the Clustree R package (version 0.5.0) (19), we assessed the cell cycle phase in the cells using the CellCycleScoring function (20) provided by the Seurat package.

### Processing of bulk RNA-seq and clinical data

We gathered cohorts from public databases including the TCGA (https://tcga-data.nci.nih.gov/tcga/), International Cancer Genome Consortium (ICGC, http://dcc.icgc.org/), ArrayExpress (https://www.ebi.ac.uk/arrayexpress/), and Gene Expression Omnibus (GEO, https://www.ncbi.nlm.nih.gov/geo/). The following criteria were employed for sample inclusion: (1) availability of survival information for at least 40 samples, (2) PDAC pathological type, (3) overall survival time exceeding 30 days, and (4) patients with primary tumors who had not undergone any prior treatment before they underwent resection. In the end, we included a total of 906 samples from 7 cohorts, which were TCGA-PDAC (140 samples), ICGC-PDAC-AU-Array (PDAC-AU-Array, 231 samples), E-MTAB-6134 (288 samples), GSE62452 (64 samples), GSE28735 (41 samples), GSE85916 (79 samples), and GSE57495 (63 samples). In addition, we enrolled 167 GTEx pancreatic tissue samples from the UCSC Xena (https://xenabrowser.net/datapages/). We also downloaded the clinical information and transcriptome data of OAK cohort (21) including 344 non-small cell lung cancer samples to assess the effect of immunotherapy. We converted the raw read count into transcripts per kilobase million (TPM) values for the GTEx and TCGA datasets. The processed expression array data obtained from ArrayExpress, GEO, ICGC, and OAK were directly generated through their respective portals. We employed bulk RNA-seq to analyze a larger number of samples for broadening our investigation of cell type composition in PDAC. The GTEx and TCGA datasets were merged to compare the abundance of Prol cells in bulk RNA-seq between PDAC and normal pancreatic samples. Genomic alterations for PDAC samples in TCGA were obtained from the cBioPortal (https://www.cbioportal.org/).

We eliminated the 7 cohorts for batch effects using z-score normalization and the surrogate variable analysis (SVA) algorithm, and we used the TCGA-PDAC cohort for training and the remaining six cohorts for testing to evaluate the predictive capacity of the Prol signature.

### Assignment of cell types for scRNA-seq cohort and identification of marker genes

To identify marker genes specific to each cell type, we applied the Wilcox test using the FindAllMarkers function in Seurat. We retained only the marker genes with an absolute log2 fold change (log2FC) of at least 0.75 and a Bonferroni-adjusted p-value of less than 0.05. The Prol marker genes, which had high expression levels (log2FC > 0.75), were then imported into the GOplot (version 1.0.2) R package for Gene Ontology (GO) annotation.

### Cell type abundance in scRNA-seq and bulk RNA-seq data

To identify cell types that were either elevated or reduced in PDAC, we conducted a Wilcoxon test between tumor and non-tumor samples using both scRNA-seq and bulk RNA-seq data. For the scRNA-seq data, we determined the observed proportions of each cell type in both tumor and non-tumor samples by dividing the number of cells belonging to a specific cell type by the total number of cells in the sample. We used the BisqueRNA R package (version 1.0.5) (22) by a PCA method to estimate the abundance of the 7 main cell types in the pancreatic bulk expression data from the TCGA cohort, which were then used for downstream analysis.

### Associations between survival outcomes and cell type proportion estimates

We conducted Cox proportional hazard regression analyses to investigate the potential associations between estimates of cell type abundance and prognosis. PDAC patients from the TCGA cohort were divided into low and high Prol cell type abundance groups using the optimal cut-off value calculated by the survminer R package (version 0.4.9). We used a Kaplan-Meier analysis based on the Log-rank test was used to compare survival differences between the two groups. Each survival analysis was carried out using the survival R package (version 3.4-0).

### Genome alterations analyses

We utilized the maftools R package (version 2.14.0) (22) to analyze and visualize genetic mutation data in the TCGA cohort. To identify the driver gene of PDAC, we applied the oncodrive module of the maftools R package, which uses a Poisson method and a P-value threshold of 0.05. This module employs the OncodriveCLUST method, which detects cancer driver genes by clustering mutations along the protein sequence. The underlying assumption is that mutations in cancer genes, especially oncogenes, tend to occur in specific protein positions that are functionally important (23). For a given gene, if a tumor sample had at least one somatic mutation type, we classified it as mutant for that gene. Conversely, if a tumor sample did not have any somatic mutation type for the gene, we classified it as wild type for that gene. The Prol cell type abundance between PDAC samples with and without somatic mutations was compared via the Wilcoxon test.

We obtained a list of oncogenes and tumor suppressor genes from the Oncology Knowledge Base (OncoKB, https://www.oncokb.org/) (24). Oncogenes or tumor suppressor genes with a copy number alteration (CNA) frequency greater than 10% were selected for conducting analyses similar to gene mutation analyses.

### Spatial transcriptomics, CellChat, CytoTRACE, and pseudotime analysis

We downloaded spatial transcriptomics (ST) data from the GEO (GSE111672). The expression profiles of the top 30 marker genes for Prol CD8+ T cells, Prol CD4+ T cells, and Prol epithelium cells were each analyzed using the GSVA (version 1.46.0) R package to calculate GSVA scores for different areas.

The CellChat R package (version 1.6.1) was utilized to conduct CellChat analysis. Initially, we segregated Prol cells from the entirety of the cells and classified them based on their cell type, including “CD8+ T”, “CD4+ T”, “B”, “Epithelium”, or “Fibroblast” cells. Following this, a CellChat object was created using the createCellChat function. For each cell signaling pathway, cell-to-cell interactions were produced utilizing the computeCommunProbPathway function.

The CytoTRACE algorithm computed a score that evaluates the differentiation and developmental potential of cells by analyzing factors such as the number of uniquely expressed mRNA features per cell and the distribution of mRNA content (25). Our study utilized the CytoTRACE R package (version 0.3.3) to determine which cell type served as the initial stage of cellular differentiation. Besides, pseudotime analysis was used to further assess the direction of cellular differentiation based on the monocle R package (version 2.24.0) (26). With the exception of q < 0.01, we maintained all other parameters at their default values.

### Response to immunotherapy and gemcitabine treatment

Tumor immune dysfunction and exclusion (TIDE) represents a highly effective approach for evaluating the immunity evasion of tumors via an examination of their expression profiles (27). Greater TIDE scores correspond to heightened potential for tumor cells to evade immune surveillance, which could potentially result in decreased efficacy of immunotherapy. In addition, the OAK cohort was used to further validate the findings of TIDE.

In TCGA drug information, each chemotherapy drug was reported with its respective response information. Specifically, we focused on samples with response information for gemcitabine chemotherapy. For samples that underwent multiple rounds of gemcitabine treatment, we only retained those with the first response information related to gemcitabine. We then assessed the correlation between Prol cell type abundance and response to immunotherapy or gemcitabine treatment.

### Signature development using unbiased machine learning-based integrative approaches

To develop an accurate and stable Prol signature, we integrated nine machine learning algorithms to construct 36 algorithm combinations. These integrative algorithms included random survival forest (RSF), elastic network (Enet), Lasso, Ridge, stepwise Cox, CoxBoost, partial least squares regression for Cox (plsRcox), supervised principal components (SuperPC), and survival support vector machine (survival-SVM). To begin with, we utilized univariate Cox regression analysis to identify prognostic markers of Prol cell type in the TCGA cohort, with a significance level of p < 0.1. Next, we employed 36 algorithm combinations on the markers to establish prediction models using 10-fold cross-validation (10-fold CV) in the same cohort. Finally, we evaluated the predictive performance of each algorithm combination by C-index across all validation cohorts, and the algorithm combination with the maximum mean C-index was chosen as the optimal one.

To screen the most valuable genes and construct the most reliable Prol signature, we employed the randomForestSRC (version 3.1.1) R package to conduct the RSF algorithm with ntree = optimal ntree and mtry = optimal mtry parameters, followed by the SuperPC algorithm. We used the superpc (version 1.2) R package for the SuperPC method, a generalization of PCA. The algorithm generates a linear combination of the features or variables of interest that captures the directions of largest variation in a dataset. The superpc.cv function was used to estimate the optimal feature threshold in supervised principal components, and it utilized a form of 10-fold CV.

### Immunohistochemical analysis and qRT-PCR

The Human Protein Atlas (HPA, http://www.proteinatlas.org/) database utilizes immunohistochemistry (IHC) techniques to provide information on protein expression in 44 major human tissues, as well as some cancer tissues. We employed the HPA database and qRT-PCR to verify the protein and mRNA expression of LY6D. We procured cell lines including the pancreas epithelial cell HPNE, pancreatic cancer BXPC-3, SW1990, PANC-1, and AsPC-1/GEM from the esteemed Shanghai Institutes for Life Sciences, affiliated with the Chinese Academy of Sciences in Shanghai, China. We propagated HPNE, BXPC-3, and SW1990 in RPMI 1640 medium supplemented with 10% FBS, penicillin (10 U/mL), and streptomycin (50 μg/mL) at 37°C in 5% CO2. We propagated PANC-1, and AsPC-1/GEM in DMEM medium supplemented with 10% FBS, penicillin (10 U/mL), and streptomycin (50 μg/mL) at 37°C in 5% CO2. We extracted RNA from both cells and tissues using Trizol reagent (Invitrogen) and performed reverse transcription with SuperScript II reverse transcriptase (Invitrogen) following the manufacturer’s protocol. Here are the primer sequences used for LY6D and β-actin:

LY6D Forward: 5’-ACTGCAAGCATTCTGTGGTCTG-3’; LY6D Reverse: 5’-CGCACAGTCCTTCTTCACCA-3’.
β-actin Forward: 5’-CACCCAGCACAATGAAGATCAAGAT-3’; β-actin Reverse: 5’-CCAGTTTTTAAATCCTGAxGTCAAGC-3.

### Statistical analysis

We used the Wilcoxon test to compare two groups and the Kruskal-Wallis test for multiple groups. Additionally, we used univariate and multivariate Cox regression analyses to identify independent predictors and performed receiver operating characteristic (ROC) analyses to assess sensitivity and specificity for survival or response prediction. We performed all statistical analyses using R software (Version 4.1.2), considering a significance level of P < 0.05.

## Results

### Detection of cell types associated with PDAC and the cell cycle using scRNA-seq data

**Figure 1:**
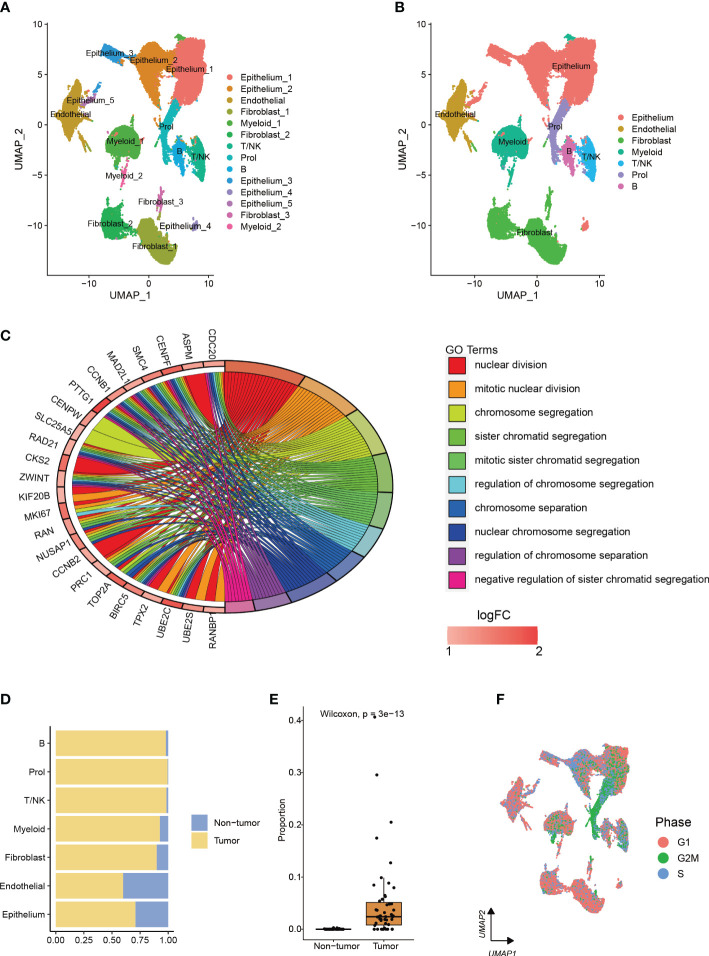
The integration of two single-cell datasets from different PDAC cohorts enables the identification and characterization of a cell type associated with PDAC. **(A, B)** The visualization of 73,415 cells using Uniform Manifold Approximation and Projection (UMAP) revealed the integration of datasets to remove the batch effect. Clusters were categorized into **(A)** 14 subtypes and **(B)** 7 major cell types. **(C)** Enrichment analysis based on upregulated marker genes of the Prol cell type. **(D)** The bar plot illustrated the cellular proportion of PDAC tumor and non-tumor samples among the full set of 73,415 cells, categorized by sub-cell type. **(E)** The Prol cell type was found to be significantly more abundant in PDAC tumor samples compared to non-tumor samples. **(F)** The cells were colored based on the inferred cell cycle phase from scRNA-seq data.

After analyzing scRNA-seq data obtained from 73,415 cells, we utilized clustering analysis to identify a total of 14 subcell types with the best resolution (resolution = 0.1) (**Figure 1A**, **Supplementary Figure 1A**). These subcell types were then grouped into 7 main cell types based on known marker genes (**Figure 1B**, **Supplementary Figure 1B**). The total number of genes identified from each cell type was shown in **Supplementary Figure 1C**. We identified a novel cell type, which comprised 99.4% of tumor cells, with high proliferation capacity, named Prol, which was enriched with genes related to cell cycle and mitosis (**Figures 1C–E**, **Supplementary Table**). To further assess the proliferation ability of Prol cells, we evaluated cell cycle scores based on the average expression of cell cycle genes. This analysis allowed us to assess the potential for cell division in Prol cells. Our results demonstrated that Prol cells exhibited significantly higher scores for the S and G2M phases in comparison to other cells (**Figure 1F**, **Supplementary Figures 1D, E**). This further suggested that Prol cell type possessed a greater capacity for proliferation than other cells. To ascertain the composition of cell types in the Prol cells, we reclassified them. In addition to epithelium cells, our analysis revealed the presence of all non-epithelium cells in the Prol cells (**Supplementary Figures 1F–L**), emphasizing the critical role of the tumor microenvironment (TME) in PDAC (28).

### The upregulated abundance of Prol Cells in PDAC correlates with unfavorable prognosis

**Figure 2:**
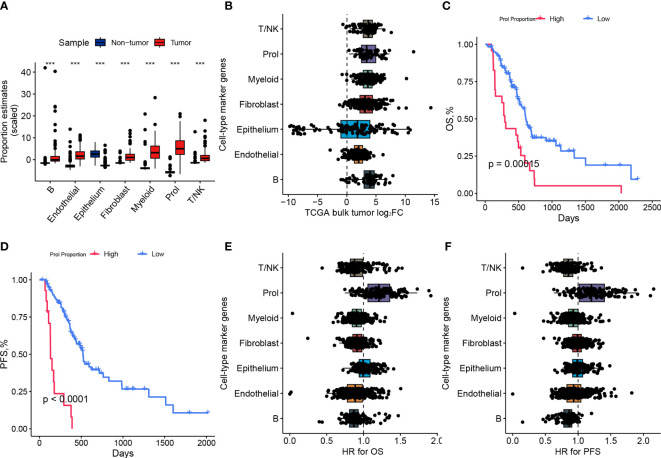
The abundance of Prol cell type was higher in PDAC and associated with a poor prognosis. **(A)** Proportions of major cell types identified in single-cell level data were estimated in pancreatic bulk RNA-seq data, followed by testing for differential abundance between tumor and non-tumor samples. **(B)** The log2 fold-changes (log2FC) of cell-type marker genes, between tumor and adjacent non-tumor samples, highlighted the association of the Prol cell type with PDAC tumors. Each dot plotted on the graph represents a gene, with its log2FC value displayed on the x-axis and its corresponding cell-type on the y-axis. **(C)** The Kaplan-Meier survival curves depicted **(C)** overall survival (OS) and **(D)** progression-free survival (PFS) in TCGA demonstrated poorer survival outcomes for patients with high frequency estimates of the Prol cell-type. **(E, F)** The association of the Prol cell-type with poor OS **(E)** and PFS **(F)** is demonstrated by the HR values for cell-type marker genes, obtained through the Cox proportional hazards regression of their expression in TCGA. Each dot plotted on the graph represents a gene, with its HR value displayed on the x-axis and its corresponding cell type on the y-axis. ***p < 0.001.

To investigate whether the changes in cell type composition that we identified in our scRNA-seq data were universally present in PDAC, we utilized bulk RNA-seq data from the TCGA and GTEx datasets to estimate the abundances of the 7 main cell types. We observed a statistically significant increase (p < 0.001) in the abundance of Prol cells in PDAC samples compared to non-tumor samples (**Figure 2A**). These findings were consistent with the observations we made in the scRNA-seq data (**Figure 1E**). We found that most of the specific Prol marker genes identified from the scRNA data with log2FC > 0.75 were highly expressed in the bulk RNA-seq data (**Figure 2B**).

We classified PDAC patients into two groups based on Prol cell abundance using the optimal cut-off value determined from the TCGA cohort to assess the prognostic significance of Prol cells. The Kaplan-Meier curve demonstrated that an upregulated abundance of Prol cells was associated with poorer overall survival (OS) and progression-free survival (PFS) (p < 0.05) (**Figures 2C, D**). We conducted a Cox regression, adjusting for B cell type abundance, T/NK cell type abundance, endothelial cell type abundance, epithelium cell type abundance, fibroblast cell type abundance, and myeloid cell type abundance, or age, gender, TMN stage, and grade, and the results showed that Prol cell abundance was an independent prognostic factor for both OS and PFS (all p < 0.05) (**Supplementary 2A–H**). Similarly, we found that the majority of Prol marker genes had the highest hazard ratio for both OS and PFS compared to all other cell types (**Figures 2E, F**) which further underscored the predictive value of Prol cells in PDAC.

### Association between somatic mutations and abundance of Prol cell type

**Figure 3:**
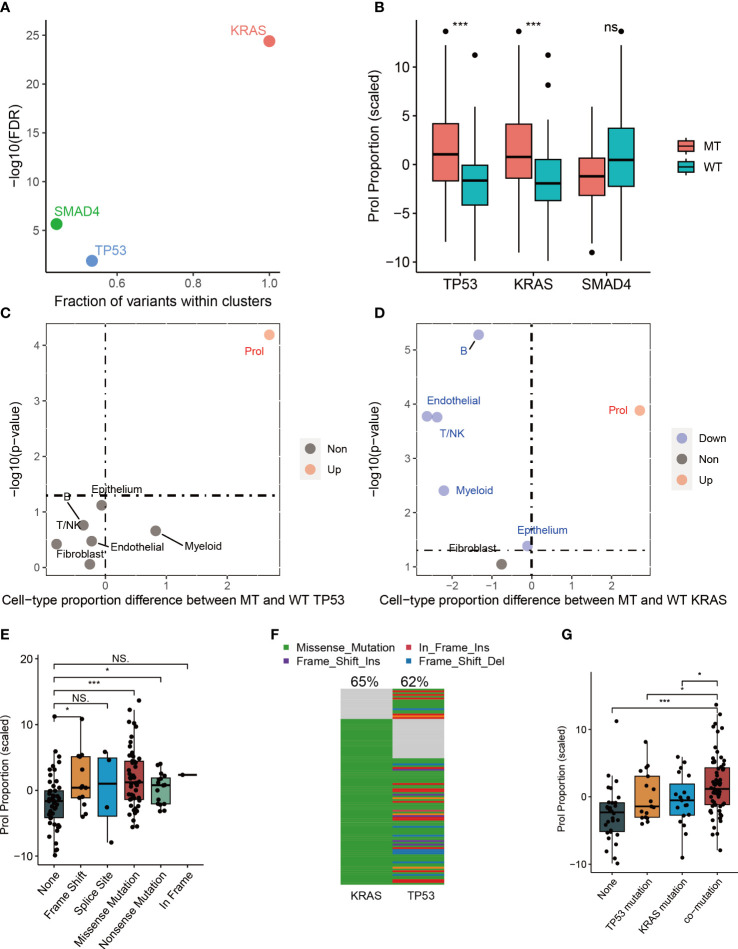
The associations between estimated cell-type proportions and somatic mutations within the TCGA cohort indicated that TP53 and KRAS mutations are linked to an increase in the abundance of the Prol cell type. The mutations demonstrated an effect on changes in the Prol cell type proportions in the bulk TCGA pancreatic samples. **(A)** The driver genes (TP53, SMAD4 and KRAS) identified by OncodriveCLUST algorithm. **(B)** The proportion estimates of Prol cell type were significantly higher in PDAC cases harboring a mutation (MT) in TP53 and KRAS compared to those with both wildtype (WT) alleles. **(C, D)** The Prol cell type was the only cell type found to be significantly increased in PDAC cases with MT TP53 **(C)** and MT KRAS **(D)**. **(E)** Proportion estimates of the Prol cell type were plotted against individuals with no TP53 mutation, and different types of TP53 mutations. Prol estimates were significantly increased in individuals with loss of function (LOF) mutations in TP53. **(F)** The mutational landscape of TP53 and KRAS. **(G)** Proportion estimates of the Prol cell type were plotted against individuals with no TP53 and KRAS mutations, single TP53 mutation, single KRAS mutation and mutations together. The significance levels for p-values in **(B, E, G)** are as follows: ^NS^p > 0.05, *p < 0.05, and ***p < 0.001.

Cancer is primarily caused by abnormal and uncontrolled cell growth due to genetic mutations (29, 30). These genetic mutations, commonly referred to as ‘drivers’ for their role in promoting tumorigenesis, give cells in somatic tissue certain selective advantages over their neighboring cells (30). Nevertheless, it is still unclear whether somatic mutations can cause specific types of tumor cells to expand or diminish. Therefore, we examined the correlation between mutations and cell type abundance in the driver genes (TP53, SMAD4, and KRAS) identified by OncodriveCLUST from the TCGA cohort (**Figure 3A**). Our findings indicated that the presence of mutant TP53 (p < 0.001) and mutant KRAS (p < 0.001) were remarkably correlated with an elevated estimated abundance of Prol cells (**Figure 3B**). It is noteworthy that Prol cells were the sole cell type that demonstrated a considerable rise in either mutant TP53 or mutant KRAS (**Figures 3C, D**).

Numerous studies have reported that mutations in TP53 can lead to a loss of the tumor suppressor function of p53, resulting in uncontrolled cell growth (31). Therefore, we further investigated the impact of different types of mutant TP53 on the abundance of Prol cells. Our findings indicated that TP53 missense, frameshift, and nonsense mutations were associated with a significantly higher abundance of Prol cells (all p < 0.05) (**Figure 3E**). Frameshift and nonsense mutations are likely to cause a complete loss of TP53 function, while missense mutations in TP53 mainly occur in the DNA-binding domain of the protein, leading to a loss of its tumor suppressor function (31). Mutations in TP53, occurring later than KRAS mutations, are found in up to 70% of PDACs and are often associated with invasive and metastatic characteristics, while also leading to gain-of-oncogenic activities (11, 32). We further explored the associations between TP53: KRAS co-mutation and the abundance Prol cells. 50.4% of PDAC patients had both TP53 and KRAS mutations (**Figure 3F**), and had a higher abundance of Prol cells (p < 0.05) (**Figure 3G**). Such patterns indicate clonal expansion of Prol cells in association with the accumulation of driver gene alterations. All in all, these findings indicate that specific somatic mutations can result in the expansion of certain types of cells, underscoring the role of KRAS and TP53 mutations in promoting uncontrolled cell growth and proliferation.

### Association between CNA and abundance of Prol cell type

**Figure 4:**
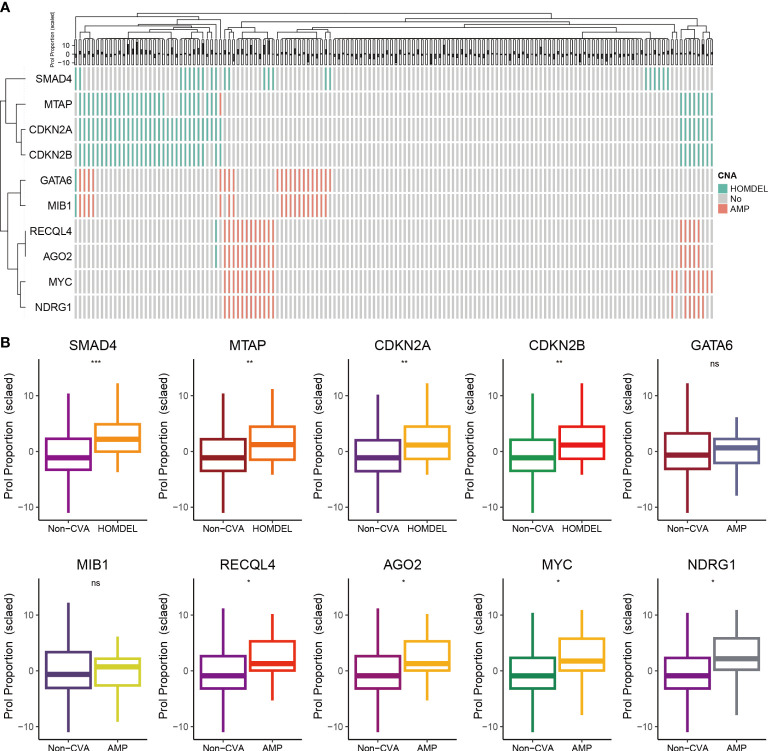
The associations between estimated Prol proportion and copy number alteration (CNA) in the TCGA cohort. **(A)** Identified oncogenes or tumor suppressor genes with CNA frequency over than 10%. **(B)** Proportion estimates of the Prol cell-type were plotted against individuals with non-CNA and homozygous deletion (HOMDEL) or homozygous deletion (HOMDEL). The significance levels for p-values in **(B)** are as follows: ^NS^p > 0.05, *p < 0.05, **p < 0.01, and ***p < 0.001.

Tumors develop as a result of the activation of oncogenes and the inactivation of tumor suppressor genes, which can occur through somatic gene mutations or CNA (33). Thus, we further explored the association between CNA landscape and the abundance of Prol cells. First, we selected oncogenes or tumor suppressor genes with CNA frequency over 10% (**Figure 4A**). Then, we found the homozygous deletion (HOMDEL) of the tumor suppressor genes SMAD4, MTAP, CDKN2A and CDKN2B were associated with higher Prol cells abundance compared with non-CNA samples, and the amplification (AMP) of the oncogene RECQL4, AGO2, MYC, NDRG1 were associated with higher Prol cells abundance (all p < 0.05) (**Figure 4B**). Overall, the amplification of oncogenes and the deletion of tumor suppressor genes may lead to higher Prol cells abundance, contributing to poor prognosis.

### Prol cell type associated with immunosuppressive and cold tumour microenvironment

**Figure 5:**
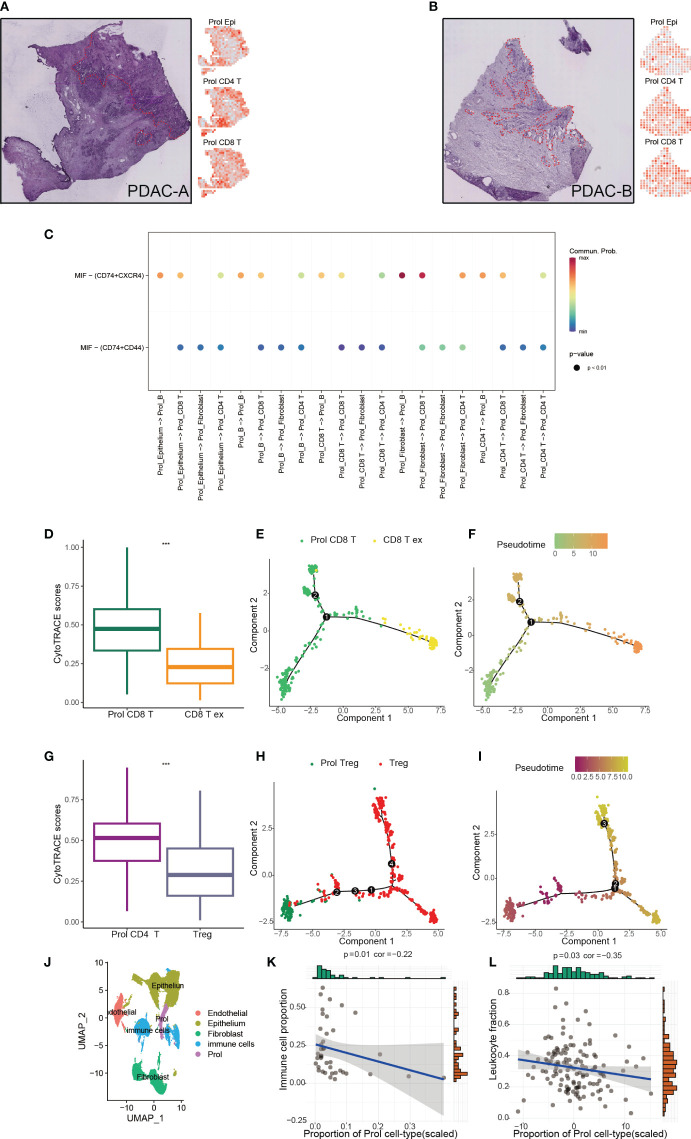
Prol cell type associated with immunosuppressive and cold tumour microenvironment. **(A)** The annotated PDAC-A tumor cryosection on the ST (Spatial Transcriptomics) slide highlighted a region with high levels of cancer cells and desmoplasia, indicated by the color red (left panel). Right panel shows Prol epi, Prol CD4+T and Prol CD4+T GSVA scores at diff ares. **(B)** The annotated PDAC-B tumor cryosection on the ST (Spatial Transcriptomics) slide highlights a region with high levels of cancer cells and desmoplasia, indicated by the color red. **(C)** MIF signaling pathway from CellChat results. **(D, G)** Box plot showed the comparison of CytoTRACE scores between Prol CD8+ T cells and exhausted CD8+ T cells **(C)**, and between Prol CD4+ T cells and regulatory T cells (Tregs) **(D)**. **(E, H)** Monocle-generated pseudotemporal trajectory of Prol CD8+ T cells and exhausted CD8+ T cells **(E)** or Prol CD4+ T cells and Tregs **(H)**. **(F, I)** Pseudotime was color-coded in different gradients from proliferating CD8+ T cells to exhausted CD8+ T cells **(F)**, and from Prol CD4+ T cells to regulatory T cells **(I)**. **(J)** T/NK cells, B cells, and myeloid cells were grouped into immune cells. **(K, L)**. Correlation between Prol cell abundance versus immune cell abundance in scRNA data **(K)** and bulk RNA data **(L)**. The significance levels for p-values in **(D, G)** are as follows: ***p < 0.001.

Apart from epithelial cells (Prol Epi), the Prol cell cluster also contained all non-epithelial cell types including CD8+ T cells (Prol CD8+ T) and CD4+ T cells (Prol CD4+ T) (**Supplementary Figure 1F**). We applied ST technology to assess the spatial locations of Prol Epi, Prol CD4+ T, and Prol CD8+ T, and found that Prol Epi, Prol CD4+ T, and Prol CD8+ T GSVA scores were all higher in the same tumor area (**Figures 5A, B**). This result indicated possible interactions between Prol Epi and both Prol CD4+ T and Prol CD8+ T. Thus, we conducted CellChat analysis, and found that the Macrophage migration inhibitory factor (MIF) pathway was active between Prol Epi and Prol CD4+ T as well as Prol CD8+ T (**Figure 5C**). In addition, we extracted T/NK cells to cluster into Treg cells, CD8+ T cells, and the other cells (**Supplementary Figures 3A–H**). Using CytoTRACE scores and pseudotime trajectory analysis, we assessed the differentiation direction for Prol CD4+ T and Prol CD8+ T. We found a higher differentiation potential for Prol CD4+ T and Prol CD+ 8 T (P < 0.05) (**Figures 5D, G**). Pseudotime trajectory analysis further demonstrated that Prol CD4+T and Prol CD8+T were in the beginning position of the differentiation process and were sequentially transformed into regulatory T cells (Treg) and CD8+ T Cell exhaustion (CD8+T ex), respectively (**Figures 5E, F**, **H, I**). Moreover, we grouped T/NK cells, B cells, and myeloid cells into immune cells (**Figure 5J**) and observed a negative association between the abundance of Prol cells and the abundance of immune cells in the scRNA-seq data (**Figure 5K**). We used Thorsson V et al.’s method, which employs DNA methylation data, to estimate leukocyte fractions from the data (34). We found that the abundance of Prol cells was negatively associated with leukocyte fractions (**Figure 5L**). These findings emphasize the critical role of Prol cells in immune evasion.

### Predictive value of Prol cells for immunotherapy and gemcitabine treatment

**Figure 6:**
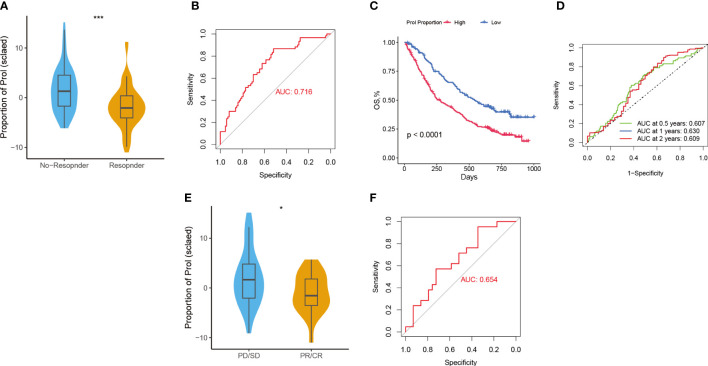
Predictive value of Prol cell-type for immunotherapy and gemcitabine treatment. **(A)** The variation in Prol cell-type abundance between TIDE prediction responders and non-responders in the TCGA cohort. **(B)** The ROC curve of Prol cell-type abundance to predict the benefits of immunotherapy in the TCGA cohort. **(C)** Kaplan-Meier curve of OS according to the Prol abundance in the OAK cohort. **(D)** ROC curve of OS according to the Prol abundance in the OAK cohort. **(E)** The variation in Prol cell-type abundance between responders and non-responders of gemcitabine treatment in the TCGA cohort. **(F)** The ROC curve of Prol cell-type abundance to predict the benefits of gemcitabine treatment in the TCGA cohort. *p < 0.05 and ***p < 0.001.

Considering that Prol cell type was associated with an immunosuppressive and cold TME, we hypothesize that PDAC patients with a higher abundance of Prol cell type may be less responsive to immunotherapy. According to the TIDE, the responder group showed a significantly lower abundance of Prol cells compared to the non-responder group (**Figure 6A**) (P < 0.05), with an area under the curve (AUC) of 0.716 for the ROC curve (**Figure 6B**). Moreover, we found that an upregulated abundance of Prol cells was associated with poorer OS in non-small cell lung cancer patients with immunotherapy (P < 0.05) (**Figure 6C**). The AUCs of the ROC curves for 0.5-, 1-, and 2-year OS were 0.61, 0.63, and 0.61, respectively (**Figure 6D**).

The responder group for gemcitabine treatment also showed significantly lower Prol cell abundance compared to the non-responder group (P < 0.05) (**Figure 6E**), with an AUC of 0.654 for the ROC curve (**Figure 6F**). In summary, these findings suggest that PDAC patients with a lower abundance of Prol cells may be more likely to benefit from immunotherapy and gemcitabine treatment.

### Prol signature generated from machine learning integrative procedures

**Figure 7:**
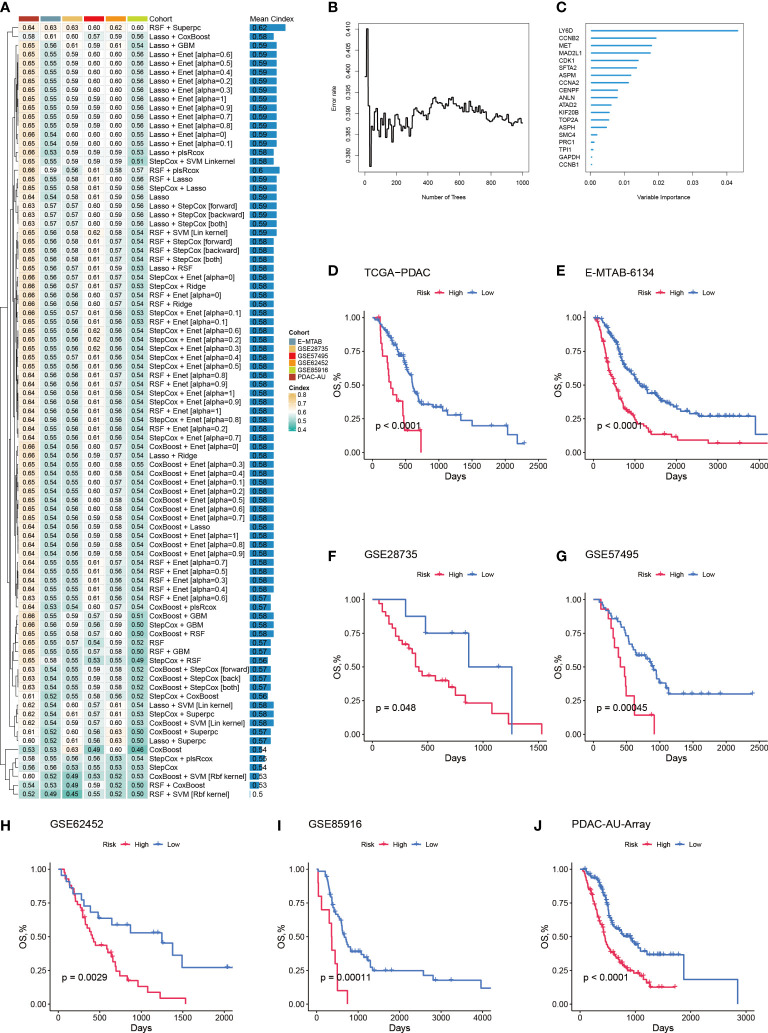
A machine learning-based integrative procedure was utilized to develop and validate a consensus Prol signature. **(A)** A total of 36 prediction models were developed using a 10-fold cross-validation framework, and the C-index of each model was computed across all validation datasets. **(B)** The number of trees determined by minimal error. **(C)** The variable importance of the 19 most valuable genes based on the random survival forest (RSF) algorithm. **(D–J)**. Kaplan-Meier curves of OS according to the Prol signature in the TCGA **(D)**, E-MATB-6134 **(E)**, GSE28735 **(F)**, GSE57495 **(G)**, GSE62452 **(H)**, GSE85916 **(I)**, and PDAC-AU-Array **(J)**.

To further quantify the abundance of Prol cells using key genes and improve the ability to predict prognosis in PDAC, we developed a Prol signature based on unbiased machine learning integrative procedures. First, univariate Cox analysis was conducted to identify 75 Prol cell type markers with prognostic significance. We used the TCGA cohort to fit 36 different algorithm combinations and calculated the C-index for each combination in the validation cohorts (**Figure 7A**). The combination of Random Survival Forests (RSF) and Superpc achieved the maximum mean C-index of 0.62 (**Figure 7A**). In the RSF, 19 genes were identified, then subjected to Superpc to construct the Prol signature (**Figures 7B, C**). After dividing the PDAC patients into high- and low-risk groups based on the optimal cutoff value of each cohort, our findings revealed that patients in the high-risk group exhibited remarkably worse OS compared to those in the low-risk group across all cohorts (all P < 0.05) (**Figures 7D–J**).

### Evaluation of the Prol signature

**Figure 8:**
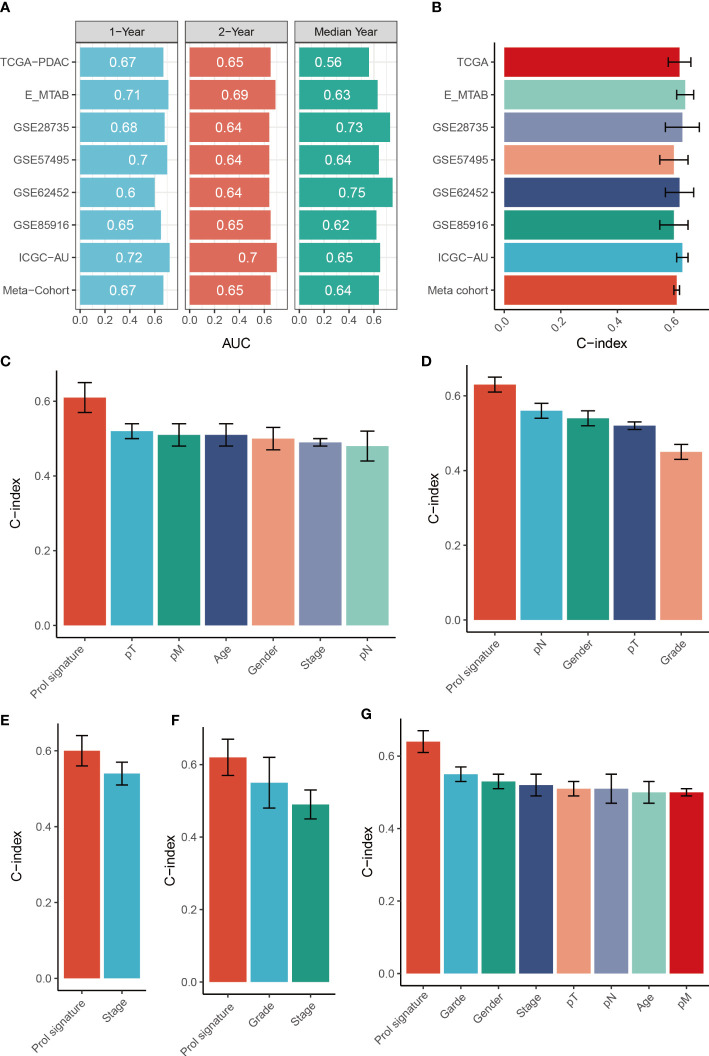
Evaluation of the Prol signature. **(A)** Time-dependent ROC analysis for predicting OS at 1, 2, and median years. **(B)** C-index of Prol signature across all datasets. **(C–G)**. The prognostic prediction performance of the Prol signature was compared with other clinical variables to predict prognosis in the different cohorts.

To assess the distinguishing ability of the Prol signature in different cohorts, we conducted ROC analyses. Our results revealed AUCs for the 1-, 3-, and median-year periods were 0.67, 0.65, and 0.56 in the TCGA-PADC cohort; 0.71, 0.69, and 0.63 in the E-MATB cohort; 0.68, 0.64, and 0.73 in the GSE28735 cohort; 0.70, 0.64, and 0.64 in the GSE57495 cohort; 0.60, 0.64, and 0.75 in the GSE62452 cohort; 0.65, 0.65, and 0.62 in the GSE85916 cohort; 0.72, 0.70, and 0.65 in the PDAC-AU-Array cohort; and 0.67, 0.65, and 0.64 in the meta-cohort, respectively (**Figure 8A**). The C-index values and their corresponding 95% confidence intervals were reported for all cohorts as follows: 0.62 [0.55–0.68], 0.64 [0.59–0.70], 0.63 [0.52–0.77], 0.60 [0.51–0.69], 0.62 [0.51–0.72], 0.60 [0.50–0.71], 0.63 [0.59–0.67], and 0.61 [0.59–0.54] (**Figure 8B**). Additionally, the OS prediction performance of the Prol signature was compared to that of other clinical features (**Figures 8C–G**). Notably, the Prol signature exhibited higher accuracy than features such as age, gender, T, N, M, grade, and stage. These findings suggest that the Prol signature is a highly reliable predictor of prognosis compared to other variables.

### Validation of expression of genes from the Prol signature

**Figure 9:**
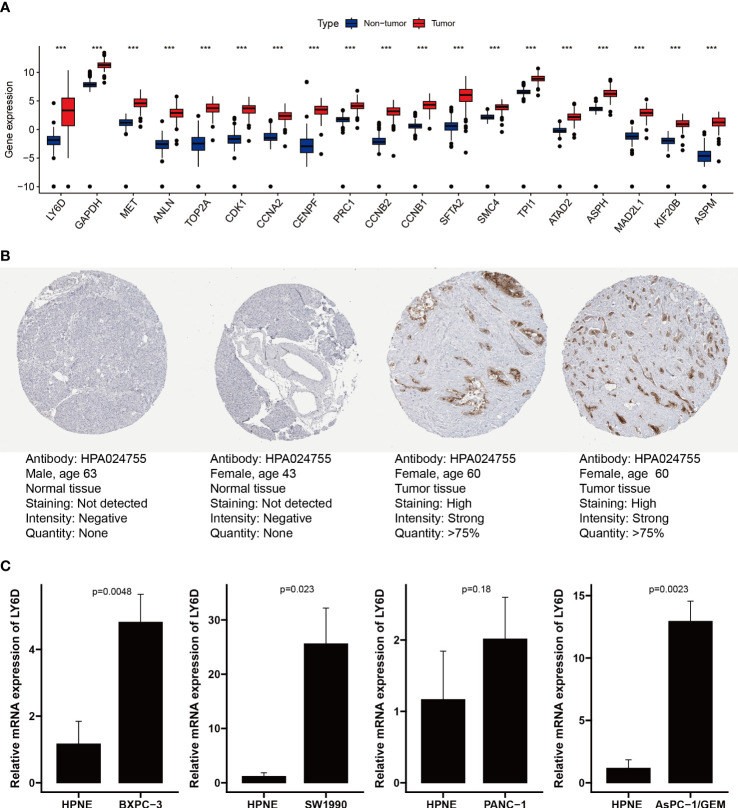
Validation of expression of genes from the Prol signature. **(A)** Differential expression of 19 model genes in normal and PDAC samples. **(B)** Immunohistochemical analysis of LY6D in normal pancreas tissue and pancreas cancer. **(C)** qRT-PCR analysis of LY6D. ***p < 0.001.

We analyzed the expression levels of the Prol signature genes in PDAC and non-tumor samples using data from the TCGA and GTEx databases. We found that all the Prol signature genes were significantly overexpressed in PDAC samples (p < 0.001) (**Figure 9A**). Among them, LY6D was the most critical gene according to the RSF. We further confirmed the mRNA and protein expression of LY6D using the HPA database and qRT-PCR. We observed that the LY6D protein expression was markedly higher in pancreatic cancer tissues than in normal pancreatic tissues (**Figure 9B**). In addition, qRT-PCR analysis showed a significant increase in LY6D expression levels in all PDAC cell lines except the PANC-1 cell line (p < 0.05) (**Figure 9C**). These results indicated that the aberrant expression of these genes, especially LY6D, may play a role in the tumorigenesis of PDAC.

## Discussion

Over the past 20 years, pancreatic adenocarcinoma incidence has gradually increased by 0.5%-1.0% per year. Despite a modest improvement in the 5-year survival rate from 5.26% to 10%, there have been no significant breakthroughs in the treatment of this disease (35). Using scRNA-seq data from PDAC and normal pancreas, we developed a new comprehensive transcriptomics framework to decompose cell types in bulk RNA-seq data of TCGA cohort. This framework has identified a proliferative cell type, Prol, that is associated with PDAC, and its high proportion in PDAC tumors is linked to significantly worse survival outcomes. The majority of marker genes specific to Prol, which were identified from scRNA-seq data, were found to be higher and associated with worse survival outcomes. Therefore, these bulk RNA-seq results obtained from scRNA-seq data further reinforce the link between Prol cell type and PDAC, as well as its correlation with worse survival outcomes, independent of the decomposition analysis. Through ST technology, cellchat analysis, and bulk RNA-seq data analysis, we found that Prol cell type was associated with immunosuppressive and cold TME. The findings of our study implied that genomic alterations were associated with the abundance of tumor-associated Prol cells in PDAC. Furthermore, we have provided evidence to suggest that PDAC patients with a lower abundance of Prol cell type may be more likely to benefit from immunotherapy and gemcitabine treatment. Finally, we have developed a Prol signature through unbiased machine learning integrative procedures to quantify the abundance of Prol cells and improve prognostic prediction based on key genes.

The TCGA project, an invaluable resource to oncologists, enabled us to identify PDAC-associated cell types and their clinical implications using our merged pancreatic scRNA-seq datasets. Although previous studies have linked several marker genes specific to Prol to poor survival outcomes (36–38), our study revealed that these genes form the Prol cells. The Prol cells comprise both previously identified PDAC genes and novel targets, including H2AFZ, HN1, and HIST1H4C, which warrant further investigation. Our approach has successfully identified more than one hundred Prol marker genes, which can provide valuable insights into the complex biological mechanisms of PDAC in future research.

KRAS activation is one of the earliest genetic events identified in PDAC, indicating the conversion of a normal centroacinar or ductal cell to an initiated cell (39). KRAS mutations are the most prevalent oncogenic alterations in PDAC, present in approximately 90% of cases, which is consistent with our results (40, 41). TP53, a tumor suppressor gene, plays a crucial role in maintaining genome integrity by regulating transcription, DNA repair, genomic stability, cell cycle control, and apoptosis (42). Previous studies have demonstrated that mutations in KRAS or TP53 induce cell cycle (42–44), which is consistent with the enrichment analysis of Prol marker genes. Our study further found that both TP53 and KRAS mutations were associated with a higher abundance of Prol cells, indicating the clonal expansion of Prol cells in association with the accumulation of driver gene alterations. In conclusion, we identified a cell type associated with somatic mutations remarkably linked to worse OS and PFS, providing novel insights into co-dependent oncological mechanisms of PDAC and strengthening current PDAC typing.

Some non-epithelial cell types were observed in the tumor-enriched Prol cells. Thus, we speculated that these non-epithelial cells may also contribute to the progression of PDAC. Based on ST technology and CellChat analysis, we found that the Macrophage migration inhibitory factor (MIF) pathway was active between Prol Epi and Prol CD4+ T as well as Prol CD8+ T. The MIF-CD74 complex triggers transcription factors that regulate cell proliferation via the ERK-MAPK and SRC pathways, which also govern gene expression (45). Besides, blocking the MIF-CD74 signaling pathway can effectively restore the antitumor immune response (46, 47). We assumed that Prol Epi might have induced T cell exhaustion via the MIF-CD74 signaling pathway. Interestingly, these results were consistent with our conjecture. Prol CD4+T and Prol CD8+T were in the beginning position of the differentiation process and were sequentially transformed into Treg and CD8+T ex, respectively. This result revealed the potential mechanisms underlying the poor prognosis associated with a high abundance of Prol cells from a different perspective. Besides, we found the abundance of Prol cells was negatively associated with immune-cell infiltration via scRNA-seq and bulk-seq data. The absence of immune cells within the tumor tissue, as indicated by a “cold” microenvironment, suggests that the tumor may not respond to immunotherapy (48, 49). Thus, we hypothesized that the high abundance of Prol cells may be associated with immunotherapy resistance, and this hypothesis was validated using TIDE. Our findings will enable individualized treatment strategies for PDAC patients and enhance their disease outcomes.

Given the dismal survival rates of patients diagnosed with PDAC, it is imperative to better understand the factors impacting survival (50). Our findings indicated that the utilization of Prol cells may have clinical significance as a prospective biomarker to aid in treatment selection and prognostication. Current clinical prognostic prediction tools for PDAC rely mainly on factors such as tumor size, invasion site, TNM stage, and the patient’s medical condition. Combining cell type markers as a method to understand cancer biology can serve as a valuable complement to existing clinical practices. The Prol marker genes have the potential to serve as a foundation for the development of novel expression-based prognostic technologies. With the maturation of RNA sequencing technology, clinical laboratories can now identify comprehensive gene expression patterns that have prognostic value (51). Thus, we developed an integrative pipeline for constructing a Prol signature by leveraging the expression profiles of Prol marker genes. Using the 10-fold cross-validation method, we fitted 36 models to the training dataset. After subsequent validation in six independent cohorts, we confirmed that the best model was a combination of RSF and Superpc. Algorithm combinations reduced the number of low-value features, optimized the model, and improved its generalization ability. The Prol signature was particularly useful for evaluating OS in PDAC. Moreover, the signature presented significantly superior accuracy than clinical traits (e.g., stage). All these suggested that the massive potential for the clinical better extrapolation and application of the Prol signature.

This study has expanded our understanding of a novel PDAC cell type with potential clinical implications. However, there are some limitations to consider. First, since our study utilized retrospective samples, future validation should be conducted in a prospective, large-scale cohort. Second, complete clinical records were not available for all patients, which could lead to bias in data analysis. Third, although we have revealed the relationship between the abundance of Prol cells and prognosis by various methods, the abundance of Prol cells has not been fully elucidated whether it is helpful for early diagnosis. Fourth, future experiments, both *in vitro* and *in vivo*, are necessary to explore the biological functions of Prol marker genes. Fifth, Superpc is a latent variable model based on linear relationships, Gaussian errors, unique principal components, and variance selection, which may not apply to some data sets with non-linear, low-variance, or multi-factor structures.

In summary, we utilized scRNA-seq data and TCGA bulk RNA-seq cohorts to decompose different cell types and identified the essential role of novel Prol cells in PDAC, as well as their impact on prognosis. The abundance of Prol cells in PDAC has been linked to genomic alterations in specific genes. Furthermore, PDAC patients with a lower abundance of Prol cells may benefit more from immunotherapy and gemcitabine treatment. Identifying tumor-associated cell types can strengthen our comprehension of cancer biology and holds significant prospects for discovering biological biomarkers in multi-center validated studies.

## Data availability statement

Publicly available datasets were analyzed in this study. This data can be found here: https://www.jianguoyun.com/p/DU0ipBUQ45m7CxiN-YgFIAA.

## Author contributions

BY, QW, and XZ drafted the initial manuscript. LZ and HL performed the literature search and collected the data. BY, YX, QL, and QZ were responsible for data analysis. SZ, TC, and JX supervised the project and designed the experiments. All the authors read and approved the final manuscript.

## Supplementary material

Supplementary Figure 1 The integration of two single-cell level datasets from different PDAC cohorts enables the identification and characterization of a cell type associated with PDAC. **(A)**. The Clustree plot was generated to identify the best resolution for the analysis. **(B)**. Dot plot displaying the percentage of cells expressing canonical marker genes and their average expression levels across 14 cell clusters. **(C)**. The total number of genes identified from each cell type. **(D, E)**. UMAP plot of 14 cell clusters, color-coded based on cell cycle scores derived from full single-cell level RNA-seq data. The plot indicated that the Prol cell cluster mainly consists of cells exhibiting elevated expression levels of **(D)** G2M phase genes and **(E)** S phase genes. **(F)**. UMAP plot of Prol cells color-coded by subcluster. **(G)**. UMAP plot depicting Prol cell colored by their main cell types. **(H-L)**. UMAP plot representing Prol cell cluster cells color-coded by their gene expression of specific marker genes, including **(H)** EPCAM (Epithelial Cells), **(I)** CD79A (B Cells), **(J)** CD8A (CD8+ T Cells), **(K)** CD4 (CD4+ T Cells), and **(L)** COL1A1 (Fibroblast), each of which is presented within individual subclusters.

Supplementary Figure 2 Univariate and multivariate Cox regression analyses of OS and PFS in the TCGA cohort. **(A–D)**. Univariate and multivariate Cox regression of OS, adjusting for B cell type abundance, T/NK cell type abundance, endothelial cell type abundance, epithelium cell type abundance, fibroblast cell type abundance, and myeloid cell type abundance **(A, B)**, or age, gender, TMN stage, and grade **(C, D)**. **(E–H)**. Univariate and multivariate Cox regression of PFS, adjusting for B cell type abundance, T/NK cell type abundance, endothelial cell type abundance, epithelium cell type abundance, fibroblast cell type abundance, and myeloid cell type abundance **(E, F)**, or age, gender, TMN stage, and grade **(G, H)**.

Supplementary Figure 3 Identification of exhausted CD8+ T cells and Treg cells. **(A)**. UMAP plot depicting T/NK cells colored by their main cell types. **(B–E)**. UMAP plot representing T/NK cell cluster cells color-coded by their gene expression of specific marker genes, including **(B, C)** CD8A and CD8B (CD8+ T Cells) and **(D)** FOXP3 (Tregs), each of which is presented within individual subclusters **(E)**. **(F, G)**. Re-grouped CD8+ T cells **(F)** to identify exhausted CD8+ T cells by their marker genes including CTLA4, TIGIT and PDCD1 **(G)**. **(H)**. UMAP plot of exhausted CD8+ T cells.
