# Supplementary figures and images for: Single-cell RNA sequencing identifies a novel proliferation cell type affecting clinical outcome of pancreatic ductal adenocarcinoma
Source: Front Oncol. 2023 Aug 2;13:1236435. doi: 10.3389/fonc.2023.1236435 (PMC10433893; doi:10.3389/fonc.2023.1236435)

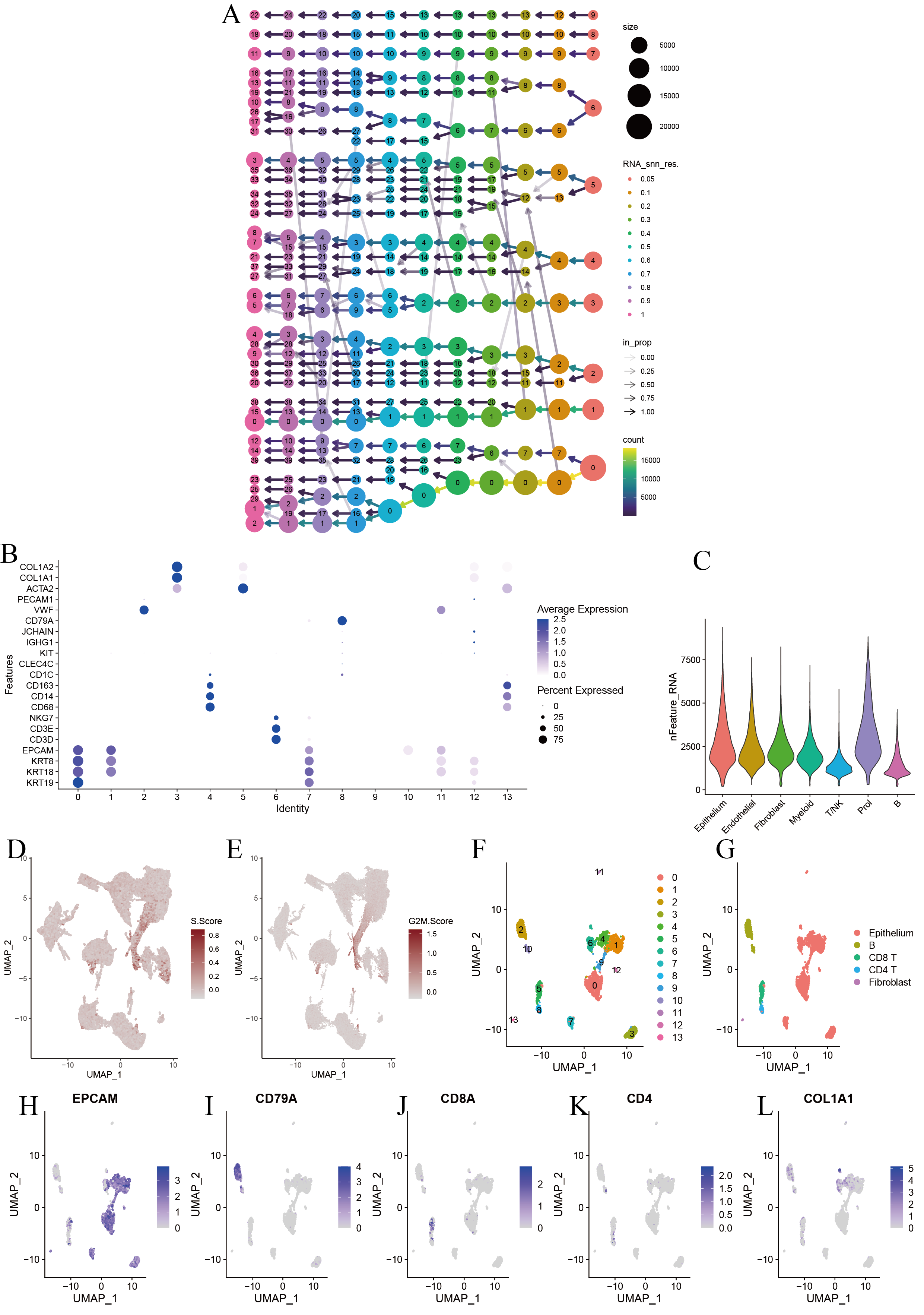

Supplement: Supplementary Figure 1 — The integration of two single-cell level datasets from different PDAC cohorts enables the identification and characterization of a cell type associated with PDAC. (A). The Clustree plot was generated to identify the best resolution for the analysis. (B). Dot plot displaying the percentage of cells expressing canonical marker genes and their average expression levels across 14 cell clusters. (C). The total number of genes identified from each cell type. (D, E). UMAP plot of 14 cell clusters, color-coded based on cell cycle scores derived from full single-cell level RNA-seq data. The plot indicated that the Prol cell cluster mainly consists of cells exhibiting elevated expression levels of (D) G2M phase genes and (E) S phase genes. (F). UMAP plot of Prol cells color-coded by subcluster. (G). UMAP plot depicting Prol cell colored by their main cell types. (H-L). UMAP plot representing Prol cell cluster cells color-coded by their gene expression of specific marker genes, including (H) EPCAM (Epithelial Cells), (I) CD79A (B Cells), (J) CD8A (CD8+ T Cells), (K) CD4 (CD4+ T Cells), and (L) COL1A1 (Fibroblast), each of which is presented within individual subclusters. [file Image_1.tif]

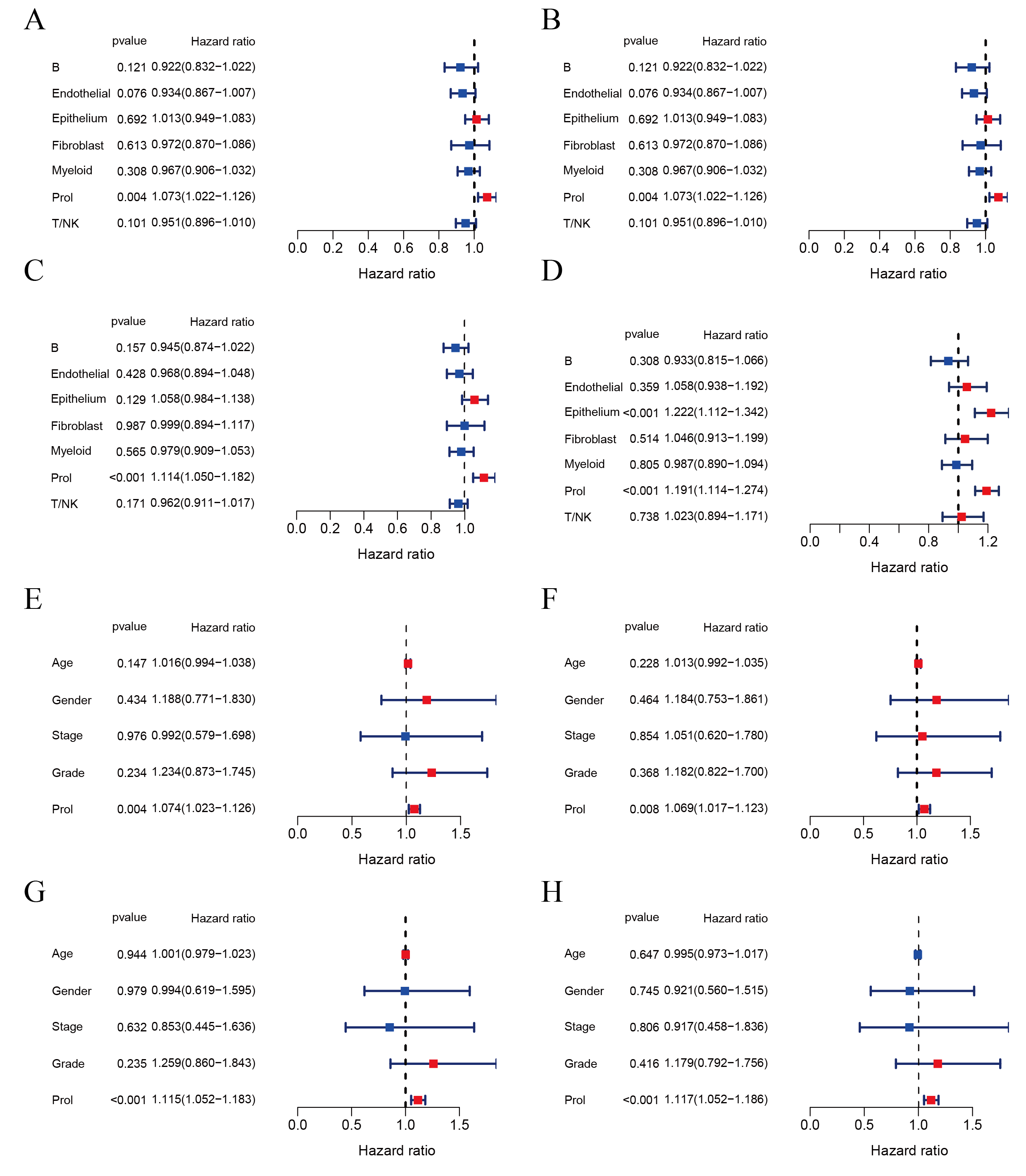

Supplement: Supplementary Figure 2 — Univariate and multivariate Cox regression analyses of OS and PFS in the TCGA cohort. (A–D). Univariate and multivariate Cox regression of OS, adjusting for B cell type abundance, T/NK cell type abundance, endothelial cell type abundance, epithelium cell type abundance, fibroblast cell type abundance, and myeloid cell type abundance (A, B), or age, gender, TMN stage, and grade (C, D). (E–H). Univariate and multivariate Cox regression of PFS, adjusting for B cell type abundance, T/NK cell type abundance, endothelial cell type abundance, epithelium cell type abundance, fibroblast cell type abundance, and myeloid cell type abundance (E, F), or age, gender, TMN stage, and grade (G, H). [file Image_2.tif]

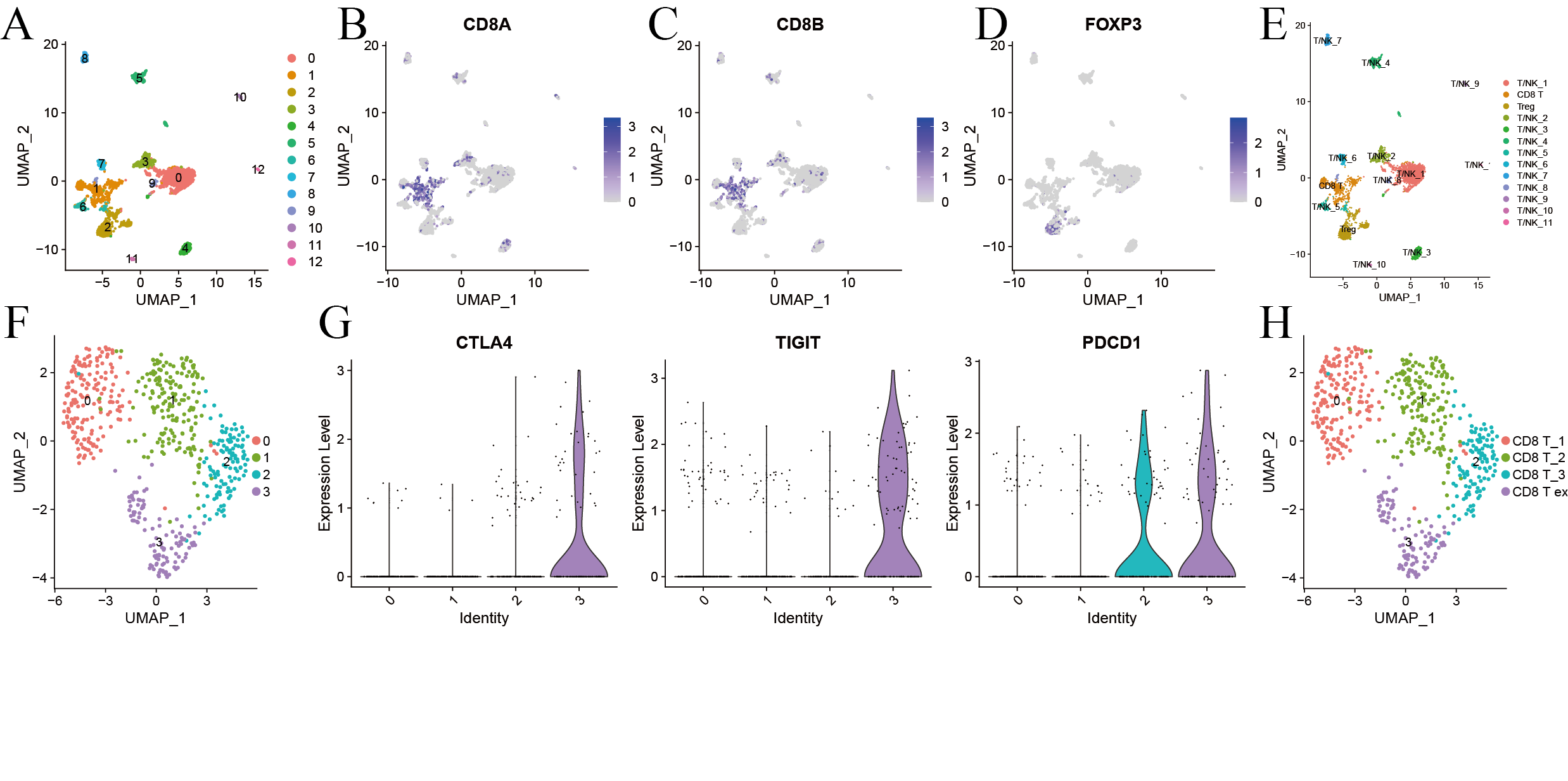

Supplement: Supplementary Figure 3 — Identification of exhausted CD8+ T cells and Treg cells. (A). UMAP plot depicting T/NK cells colored by their main cell types. (B–E). UMAP plot representing T/NK cell cluster cells color-coded by their gene expression of specific marker genes, including (B, C) CD8A and CD8B (CD8+ T Cells) and (D) FOXP3 (Tregs), each of which is presented within individual subclusters (E). (F, G). Re-grouped CD8+ T cells (F) to identify exhausted CD8+ T cells by their marker genes including CTLA4, TIGIT and PDCD1 (G). (H). UMAP plot of exhausted CD8+ T cells. [file Image_3.tif]
